# Schistosomiasis among Travelers: New Aspects of an Old Disease

**DOI:** 10.3201/eid1211.060340

**Published:** 2006-11

**Authors:** Eyal Meltzer, Galit Artom, Esther Marva, Marc Victor Assous, Galia Rahav, Eli Schwartz

**Affiliations:** * The Chaim Sheba Medical Center, Tel Hashomer, Israel;; † Sackler School of Medicine, Tel Aviv University, Tel Aviv, Israel;; ‡ Maccabi Healthcare Services, Tel Aviv, Israel;; § Central Laboratories Ministry of Health, Jerusalem, Israel

**Keywords:** Schistosomiasis/diagnosis, schistosomiasis/therapy, Schistosoma haematobium, Schistosoma mansoni, travel, research

## Abstract

Schistosomiasis is increasingly encountered among travelers returning from the tropics; signs and symptoms of travelers may differ from those of local populations. During 1993–2005, schistosomiasis was diagnosed in 137 Israeli travelers, most of whom were infected while in sub-Saharan Africa. Clinical findings compatible with acute schistosomiasis were recorded for 75 (66.4%) patients and included fever (71.3%), respiratory symptoms (42.9%), and cutaneous symptoms (45.2%). At time of physical examination, 42 patients (37.1%) still had symptoms of acute schistosomiasis, chronic schistosomiasis had developed in 23 (20.4%), and 48 (42.5%) were asymptomatic. Of patients who were initially asymptomatic, chronic schistosomiasis developed in 26%. Diagnosis was confirmed by serologic testing for 87.6% of patients, but schistosome ova were found in only 25.6%. We conclude that acute schistosomiasis is a major clinical problem among travelers, diagnostic and therapeutic options for acute schistosomiasis are limited, and asymptomatic travelers returning from schistosomiasis-endemic areas should be screened and treated.

Schistosomiasis is a common parasitic infection in the developing world, especially in Africa, where it is hyperendemic in many regions. In Israel, schistosomiasis is no longer endemic; however, cases have been reported in travelers returning to Israel. Research on the clinical, diagnostic, and therapeutic aspects of chronic infection in disease-endemic populations is extensive; however, data about schistosomiasis in travelers returning from these areas are relatively sparse. Moreover, because local inhabitants of disease-endemic areas are exposed repeatedly, the clinical manifestations may differ between residents and visitors. We examined the epidemiology and clinical manifestations of schistosomiasis in travelers returning to Israel.

## Methods

We used a list of all laboratory-confirmed diagnoses of schistosomiasis in Israelis from the Ministry of Health in Jerusalem, Israel, and the Centers for Disease Control and Prevention (CDC) in Atlanta, Georgia, USA, during 1993–2005. Schistosomiasis is reportable in Israel, and all diagnoses of schistosome ova in stool or urine in patients should be referred to the central laboratory of the Ministry of Health. Before August 2003, serologic tests were mostly referred to CDC for fast-ELISA screening with Schistosoma mansoni adult microsomal antigen and for confirmation and speciation by enzyme Western blot, which has a sensitivity and specificity of ≈100% (*1*). Since then, serologic diagnosis of schistosomiasis has become available at the Ministry of Health and uses a soluble egg antigen ELISA (IVD Research Inc., Carlsbad, CA, USA) that is not species specific.

Clinical and epidemiologic data were available for all cases diagnosed and patients treated at the Center for Geographic and Tropical Medicine at Sheba Medical Center. If data were incomplete, patients were contacted by phone for additional details. We examined only cases in Israeli travelers; cases in immigrants from disease-endemic countries, who may have been previously exposed to Schistosoma, and foreign workers were excluded. The study was approved by the ethical committee of Sheba Medical Center.

Data were analyzed according to the presence of symptoms suggestive of acute schistosomiasis, the evolution to chronic schistosomiasis (genital, urinary, or gastrointestinal), or the diagnosis of asymptomatic schistosomiasis through screening. A case of acute schistosomiasis was defined as the onset of fever or hypersensitivity symptoms—urticarial rash, angioedema, dry cough or wheeze—after exposure to infected water sources, with subsequent confirmation by serologic or parasitologic testing. A source of infection was defined as self-reported exposure to fresh water (e.g., bathing, diving) in a schistosomiasis-endemic region. Exposure time was measured from the first day of exposure to the last. The time from exposure to illness was measured from the last exposure to the onset of symptoms. The Fisher exact test and Student t test were used to analyze qualitative and quantitative variables, respectively; level of significance was set at p<0.05.

## Results

During 1993–2005, a total of 137 travelers returning to Israel had a laboratory diagnosis of schistosomiasis. The mean annual number of cases was 10.5 (range 1–13 cases/year) with the exception of 1998, when 27 cases associated with rafting trips to the Omo River in Ethiopia were diagnosed (*2*). The mean ± SD age of the travelers was 27.1 ± 6.8 years, and the male:female ratio was 2.42:1. S. haematobium caused 39.4% of cases; S. mansoni caused 29.9%, a mixed infection caused 16%, and undetermined species (because the test was not species specific) caused 14.7%. One patient was seropositive for S. mekongi.

Epidemiologic data were available for 113 (82.5%) patients. All infections were acquired in Africa except 1, which was acquired by a traveler who had bathed in the Mekong River in East Asia. Twenty-five (22.1%) patients were exposed to 2 potentially infected water sources. The other patients were exposed to only 1 source, mostly Lake Malawi (57%) and the Omo River in Ethiopia (18%). The median exposure time was 2 weeks (interquartile range 1.5–3.0 weeks).

Clinical data were available for 113 patients, of whom 104 were evaluated at the Sheba Medical Center (Table 1). Of these, 48 (42.5%) were asymptomatic at the time of evaluation, and the rest had symptoms of either acute or chronic schistosomiasis (Figure); demographics and duration of travel were similar regardless of the symptom phase at evaluation (Table 1). Immediate symptoms after bathing (acute itching and rash, suggestive of swimmer's itch) were reported by only 8 (7.1%) patients.

**Table 1:** Clinical and laboratory data for patients with schistosomiasis, by stage of disease, Israel, 1993–2005

	Acute (n = 42)	Chronic (n = 23)	Asymptomatic (n = 48)	p value
Age ± SD, y	28.1 ± 8.0	25.1 ± 2.6	27.2 ± 6.8	NS*
Male:female ratio	2.63:1	4.5:1	2.5:1	NS
Exposure, weeks ± SD	2.3 ± 1.9	4 ± 5.8	2.1 ± 0.8	NS
Exposure to symptoms, weeks ± SD	3.1 ± 2.7	58.0 ± 31.5	–	<0.001†
Eosinophil count, ×10^9^/L ± SD	2,374 ± 1,937	864 ± 529	1,363 ± 1,490	<0.05† NS‡
*Schistosoma haematobium§*	42.5%	60.9%	27.1%	NS
*S. mansoni§*	27.5%	0%	52.1%	<0.05†‡
Ova detection	25%	56.5%	14.6%	<0.02† NS‡
Serologic diagnosis	95%	96%	89.5%	NS

*NS, not significant. †Comparison between acute and chronic schistosomiasis. ‡Comparison between acute and asymptomatic schistosomiasis. §In 14.7% of all cases, the test used was not species specific.

**Figure:**
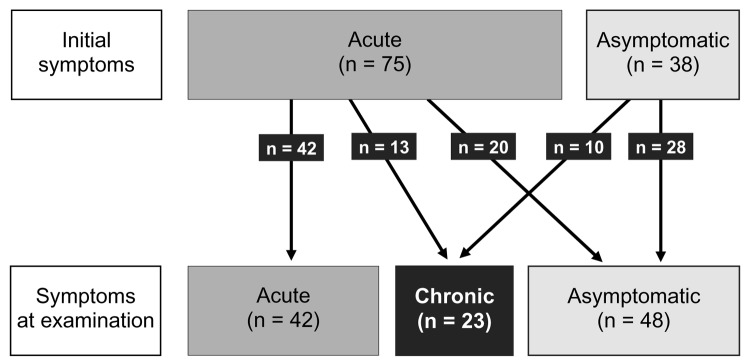
Progression of schistosomiasis symptoms among Israeli travelers (n = 113), 1993–2005.

### Acute Schistosomiasis

Of the 42 (37.2%) patients with acute schistosomiasis, symptoms occurred after a median of 3 weeks (mean ± SD 3.1 ± 2.7 weeks) from exposure. Fever was the prevalent symptom, followed by respiratory symptoms and rash. Only 4 (9.5%) patients had the complete complex of acute schistosomiasis: fever, urticaria, and respiratory symptoms. An additional 33 patients reported having had an episode of symptoms after exposure that was compatible with acute schistosomiasis but resolved before medical examination (Table 2). For most patients, the duration of fever and urticaria was short, usually 1–2 weeks (range 1–6 weeks), and the median duration of respiratory symptoms was longer, 6 weeks (mean ± SD 15.4 ± 22.7 weeks).

**Table 2:** Symptoms reported by patients with acute schistosomiasis, Israel, 1993–2005

Symptom	During examination (n = 42), no. (%)	During examination and by history (n = 75), no. (%)
Fever	30 (71.3)	51 (68.0)
Respiratory*	18 (42.9)	30 (45.0)
Cutaneous†	19 (45.2)	23 (30.7)
Combinations		
Fever and respiratory	6 (14.2)	11 (14.7)
Fever and cutaneous	11 (26.2)	13 (17.3)
Fever, cutaneous, and respiratory	4 (9.5)	5 (6.7)

*Cough, wheeze, dyspnea. †Pruritus, urticaria, angioedema.

### Chronic Schistosomiasis

Of the 23 (20.3%) patients with chronic schistosomiasis, time from exposure to examination ranged from 4 months to 3 years (mean ± SD 58.0 ± 31.5 weeks). Among them, 13 had a history of an acute schistosomiasis-like illness, 21 (91.3%) had genitourinary symptoms (17 hematuria or dysuria, 4 hematospermia), 2 had gastrointestinal symptoms, and 1 had protracted fatigue and abdominal pain. No patient had renal failure, obstructive uropathy, cirrhosis, or central nervous system involvement; however, 7 (30.4%) patients underwent invasive diagnostic procedures for suspected tumors of the bladder or colon, and their travel history was considered only after the diagnosis of chronic schistosomiasis had been made.

### Asymptomatic Schistosomiasis

A total of 48 (42.5%) patients were examined while asymptomatic. These patients included 5 who were referred because of eosinophilia and 5 who were hospitalized in Israel because of malaria and had serologic testing because of their travel history. The rest of the patients came in for screening because schistosomiasis had been diagnosed in a traveling companion or because they had been exposed to fresh water while traveling. A history of an acute schistosomiasis-like illness was elicited from 20 of these asymptomatic patients.

### Laboratory Findings

For patients with acute schistosomiasis, physical examination findings were usually unremarkable. Laboratory test results showed eosinophilia in 73% and mildly abnormal liver function in 17%. Eosinophilia was more prevalent in patients with acute than with chronic schistosomiasis (Table 1).

Diagnoses were confirmed by serologic testing for 120 (87.6%) patients. For only 35 (26.9%) patients, mostly those with chronic schistosomiasis, were schistosome ova found in urine, semen, or stool samples (Table 1). For 6 patients, the diagnosis was established by chance in a tissue biopsy; subsequent testing showed ova in the stool or urine of 5 of these 6 patients. A diagnosis of schistosomiasis was made for 1 additional patient, whose colectomy specimen (colectomy performed because of familial adenomatous polyposis) contained schistosome ova 10 years after exposure (*3*).

### Treatment

Praziquantel was offered to all 113 patients, but follow-up information after treatment was available for only 65. Eosinophil counts declined from a mean ± SD of 2,100 × 10^9^/L ± 1,850 to 970 × 10^9^/L ± 1,575 (p<0.01). Because respiratory symptoms were the least self-limiting form of acute schistosomiasis, response to therapy was evaluated in these patients. Among the 38 patients with respiratory symptoms, 4 cases resolved before praziquantel therapy, and follow up was incomplete for 12. Of the remaining 22 patients, in 9 cases there was an acute exacerbation of symptoms after praziquantel therapy, lasting from a few days to 3 weeks. Corticosteroids were prescribed for 5 patients with protracted respiratory symptoms, and repeated courses of praziquantel were prescribed for 6. Overall, in 20 of 22 cases, symptoms improved within 2 months after therapy. A history of asthma was not associated with a worsening of symptoms after therapy.

## Discussion

Although schistosomiasis is widespread throughout much of the tropics, we found that among Israeli travelers, schistosomiasis is acquired almost exclusively in Africa. This finding supports data from the GeoSentinel group and the TropNetEurope surveillance network, which report that Africa was the source for ≈90% of all cases (*4**,**5*). The absence of cases from other disease-endemic regions may be because schistosomiasis is often a focal disease, and perhaps infections are not commonly found in the specific locations frequented by travelers in South America and the Far East.

The schistosome life cycle has 3 phases in the human host, and all have their clinical counterparts. The first phase is host penetration by cercaria, which is usually manifested as a transient (hours) itchy eruption that occurs soon after exposure and is known as swimmer's itch or cercarial dermatitis. The second phase is schistosomulae tissue migration and maturation and is associated with transient (days to weeks) hypersensitivity, which gives rise to the syndrome of acute schistosomiasis. The third phase, endovascular egg production, is protracted (years) and is associated with organ damage (genitourinary, gastrointestinal, or ectopic migration to other organs).

In our cohort, a history suggestive of the first phase, swimmer's itch, was infrequent, found in 7.1% of patients. Other case series also suggest that it occurs in only 12%–36% of patients with schistosomiasis (*6**–**8*). For many of our patients, data were collected long after exposure, so they may have forgotten such a transient phenomenon. Obviously, the absence of a history of swimmer's itch cannot be used to exclude schistosomiasis.

Our data show acute schistosomiasis, the second phase, to be the predominant form of the disease in travelers, noted by 66.4%. Travelers in that respect are markedly different from local populations and immigrants, among whom acute schistosomiasis is rare. Some textbooks suggest that acute schistosomiasis is a syndrome that typically results from heavy infestations with S. japonicum or S. mansoni and rarely, if ever, from S. haematobium (*9*). Our findings suggest that, to the contrary, acute schistosomiasis occurs with S. haematobium and with S. mansoni. We believe that acute schistosomiasis is an immune phenomenon, is not species specific, and can develop after infection with each of the schistosomae that infect humans. That some species (e.g., S. mekongi and S. interacalatum) are rarely reported in the literature as causing acute schistosomiasis may merely reflect lack of traveler exposure.

The acute symptom complex that appears several weeks after exposure is often called Katayama fever, having been described in Katayama district near Hiroshima in Japan in 1847 (*10*). However, our data show that fever occurs in only 71% of patients with acute schistosomiasis; similarly, data from the early 20th century show that fever was not universally present during the acute toxemic phase (*11*). We therefore believe that acute schistosomiasis, rather than Katayama fever, should be the preferred name for this syndrome.

Although fever is indeed not universal with acute schistosomiasis, when it does occur, it is usually high; therefore, malaria is a major differential diagnosis. This may help explain why most patients evaluated abroad were treated for malaria. Antimalarial drugs were offered to some patients despite negative smear results. In other patients reported to have positive malaria smear results in Africa, serologic testing for plasmodia after their return was found to be negative, which adds to reports of the unreliability of tests performed in Africa (*12*). For other patients, fever and urticarial rash were diagnosed as allergic reactions and treated with glucocorticoids. Urticarial rash or eosinophilia are clinical clues that may help differentiate between the fever of acute schistosomiasis and that of malaria. Also, persistent fever despite documented parasitologic response in cases of proven malaria should alert the clinician to the possibility of coinfection.

The 3 major clinical features—fever, skin, and respiratory symptoms—occur in combination in only a small percentage of patients with acute schistosomiasis (Table 2). Even then, they often do not appear simultaneously. Unfortunately, in our experience, even when returning travelers had a history of the complete symptom complex, the clinical diagnosis was frequently missed. The respiratory symptoms tend to follow a more protracted course, and unless a thorough history is taken, the case may be misdiagnosed as allergy or asthma.

In chronic schistosomiasis, the third phase, genitourinary problems and, less often, gastrointestinal problems occur. Late sequelae such as cirrhosis, portal/pulmonary hypertension, or obstructive uropathy are the aftermath of heavy or repeated infection typical for local populations; absence of these sequelae among travelers is not surprising. Of seropositive patients who were initially asymptomatic, chronic schistosomiasis developed in 26% (Figure). The question of whether asymptomatic travelers with history of freshwater exposure should be screened and treated for schistosomiasis is clearly pertinent (*2*). Our data strongly support the recommendation to screen and treat.

Our study illustrates the problems encountered in diagnosing Schistosoma infection in patients evaluated during acute schistosomiasis. Stool and urine examinations for ova were largely negative, diagnoses relied on serologic testing, and therefore most diagnoses were made post facto. Although serologic testing appears to be very sensitive, a caveat must be added that most serum samples were collected relatively late in the course of acute schistosomiasis. For the acutely ill febrile returning traveler, serologic test results may be negative, as occurred for 2 of our patients for whom an additional specimen was required for diagnosis.

The ability to diagnose chronic schistosomiasis by ova detection was higher (Table 1); however, for many patients, only serologic testing gave a positive result. Thus, ova detection, which is the main diagnostic tool in schistosomiasis-endemic countries, often gives negative results for travelers. Negative results occur during the acute stage because symptoms start before oviposition and during the chronic stage because of low-level or intermittent oviposition. Successful ova detection entails multiple examinations of stool or urine; sensitivity depends greatly on technique and expertise, which may be declining at many Western institutions.

Treatment of schistosomiasis can also be problematic. Praziquantel is considered the drug of choice for schistosomiasis but possesses little activity against the juvenile forms of the helminth (schistosomulae) (*13*). Of our patients who were exposed in Lake Malawi, 11 took praziquantel soon after exposure while abroad, as a form of preemptive therapy for later schistosomiasis, which is a common recommendation for visitors to Lake Malawi. Of these, acute schistosomiasis developed in 7, symptoms lasted 5–45 weeks, and chronic schistosomiasis developed in 2. These cases show that praziquantel given shortly after exposure does not preclude later clinical infection.

The limitations of praziquantel for treating acute schistosomiasis were clearly seen in our patients. For those who had fever and rash, symptom duration was relatively short, and the contribution of therapy is unclear. For patients with respiratory symptoms, therapy was sometimes associated with an acute exacerbation of symptoms. For some patients, cough and dyspnea continued for many months after treatment. Our policy is to repeat praziquantel treatment after 3 months, which may be why we did not see chronic schistosomiasis in this group.

## Conclusions

Most cases of schistosomiasis in Israeli travelers are acquired in sub-Saharan Africa. For travelers, most symptomatic cases of schistosomiasis are acute, diagnosis by ova detection is usually negative, and serologic testing, when performed too early, may give false-negative results. Modern practitioners may not be in a better situation than those who treated acute schistosomiasis cases in the 1920s (*11*). Our findings lend support for screening asymptomatic travelers to schistosomiasis-endemic areas because chronic schistosomiasis will develop in many of them. Better knowledge of the various possible manifestations of schistosomiasis should decrease the time to diagnosis of acute and chronic schistosomiasis. Physicians should promptly evaluate returning travelers for schistosomiasis even if they do not report the complete symptom complex. However, in the absence of effective treatment for acute schistosomiasis, new diagnostic methods and new drugs that affect the parasite at an early stage are needed.
